# Immunogenomic Profiling and Classification of Prostate Cancer Based on HIF-1 Signaling Pathway

**DOI:** 10.3389/fonc.2020.01374

**Published:** 2020-08-06

**Authors:** Jukun Song, Weiming Chen, Guohua Zhu, Wei Wang, Fa Sun, Jianguo Zhu

**Affiliations:** ^1^Medical College, Guizhou University, Guiyang, China; ^2^Department of Oral and Maxillofacial Surgery, Guizhou Provincial People's Hospital, Guiyang, China; ^3^Department of Urology, Guizhou Provincial People's Hospital, Guiyang, China

**Keywords:** prostate cancer, tumor immunity, immunogenomic profiling, classification, HIF-1 signaling pathway

## Abstract

The HIF-1 signaling pathway plays an important role in the pathogenesis of cancer. Many studies have explored the progression of prostate cancer (PCa) under hypoxic conditions based on transcriptome data; few have uncovered the immunogenomic profiling and prostate cancer classification based on the HIF-1 signaling pathway. This pathway may help to identify the optimal subset of PCa patients responsive to immunotherapy/chemotherapy. The immunogenomic PCa subsets were classified based on profiling of the HIF-1 signaling pathway, using four publicly available PCa datasets. Three PCa subtypes that named as HIF-1 High (HIF-1_H), HIF-1 Medium (HIF-1_M), and HIF-1 Low (HIF-1_L) were identified. Functional enrichment was analyzed in each subtype. Several cancer-associated and immune-related pathways were hyperactivated in the HIF-1_H subtypes. In contrast, HIF-1_L subtypes were enriched in cell cycle and cell repair. Compared with other subtypes, HIF-1_H subtypes have greater immune cell infiltration, anti-tumor immune activity, and better survival prognosis. The submap and TIDE algorithm were used to predict the clinical response to immune checkpoint blockade, and GDSC was employed to screen potential chemotherapeutic targets for the treatment of PCa. Several chemotherapy drugs were identified in the GDSC dataset, including ABT 888, Temsirolimus, and EHT 1864. Meanwhile, HIF-1_H was defined as an early PCa marker, which is more likely to respond to immunotherapy. The identification of immunogenomic PCa subtypes based on the HIF-1 signaling pathway has potential clinical implications for PCa treatment. Immunopositive PCa subtypes will help to explore the reasons for the poor response of PCa to immunotherapy, and it is expected that immunotherapy will guide the personalized treatment of PCa patients.

## Introduction

Prostate cancer (PCa) remains the most common malignancy in western countries. In America, PCa deaths ranked second to breast cancer (1). The American Cancer Society announced 174,650 new cases of PCa in 2019, ranking first at 20% of new male cancer cases, with 31,620 deaths accounting for 10% of total cancer deaths (2). Prostate cancer is the major tumor type in 28 European countries, and the second most prominent type in seven other countries (3). The ethnic differences in the incidence of prostate cancer are distinct. The incidence and mortality rate of PCa in China are lower than in Western countries, such as Europe and the United States. However, with the advancement of society and changes in people's lifestyles, PCa has become a common tumor in the male urinary tract, and its incidence has increased annually (4). Prostate cancer is a heterogeneous disease that can vary greatly even within the same tumor (5). Early treatment of PCa using androgen deprivation therapy has achieved early satisfactory results, but ultimately inevitable to progress to hormone-dependent PCa, which causes clear clinical symptoms (6). The cancer phenotype is not only defined by the intrinsic activity of the tumor cells but also by immune cells recruited to its microenvironment. The role of immune cells in the tumor-associated microenvironment during tumor development has not been fully understood, especially in PCa.

Hypoxia-inducible factor-1 (HIF-1) is a major regulator of the cell's response to a hypoxic microenvironment, which is strictly controlled through synthesis, and degradation (7). Hypoxia and overexpression of HIF-1 may be related to radiotherapy and chemotherapy resistance, increased risk of tumor invasion and metastasis, and poor clinical prognosis of most solid tumors, especially PCa (8); therefore, the HIF_1 pathway is considered as a viable pharmacological target in the treatment of solid tumors (9, 10). Hypoxia has been linked to cancer progression, recurrence, and metabolic reprogramming. Under hypoxic conditions, HIF-prolyl hydroxylases (PHDs) activity is inhibited, HIF-1a accumulates, and dimerizes with HIF-1b, thereby activating transcription of hundreds of genes. The prevalence of hypoxia and the increase in HIF-1α have raised interest in targeting the HIF pathway for most solid tumors. Recent evidence from genetic and pharmacological research supports the view that inhibition of HIF-1 is beneficial for cancer treatment (11–13).

Cancer has gradually been recognized as an adaptive and complex system, and it is increasingly difficult to achieve the desired therapeutic effect using most single-target drugs. Immunotherapy is a promising therapeutic option for cancer, that also prevents drug resistance. It has achieved satisfactory results for some types of cancer, such as malignant melanoma, but is not effective in the treatment of PCa (14, 15). Specific genetic or genomic features, such as tumor mutation burden (TMB), neoantigen load, PD-L1 expression, and deficient DNA mismatch repair, have been associated with cancer immunotherapeutic response. Three immunogenomic PCa subtypes were classified based on HIF pathway enrichment scores by transcriptome data. The stability and reproducibility of this classification were validated in three other independent datasets. This study identified the subtype-specific molecular features, including genes, gene ontology, pathways, and networks. We found a subtype of immunopositive PCa subtype which will help to explore the reasons for the poor response of PCa to immunotherapy; it is expected that immunotherapy will be used in the individualized treatment of PCa patients.

## Methods

### Data Sources

Gene expression profiles were downloaded from three publicly available datasets: Taylor (16), TCGA (17), and two GEO datasets [GSE70768 (18) and GSE68555] (19). The TCGA dataset included 499 tumor samples, Taylor dataset enrolled 150 samples, and GSE70768 and GSE68555 datasets had 125 and 128 tumor samples, respectively. The RNA-seq profiles and phenotype data were downloaded. The expression matrix and clinical characteristics of each patient were collected manually. Patients with full clinical data and survival time of more than 30 days were included in the study.

### Gene Set Variation Analysis (GSVA) and Unsupervised Clustering Analysis

The Gene set variation analysis (GSVA) was employed to derive the absolute enrichment scores, to calculate HIF-1 signaling pathway enrichment in PCa samples (20). First, the gene set of the HIF-1 signaling pathway (hsa04066) was downloaded from KE (Kyoto Encyclopedia of Genes and Genomes) dataset (http://www.genome.jp/kegg/) (Supplementary Table 1). Then, GSVA was used to analyze the enrichment scores based on the HIF-1 signaling pathway in different PCa samples. The hierarchical clustering of PCa samples was done based on the enrichment scores of the HIF-1 signaling pathway.

### Implementation of Single-Sample Gene Set Enrichment Analysis (ssGSEA)

The ssGSEA predicted 29 immune cells that are involved in innate and adaptive immunity, using gene signatures expressed by immune cell populations of individual PCa samples (21, 22). The enrichment scores of the 29 immune signatures were quantified by ssGSEA for each PCa dataset.

### Assessment of Immune Cell Infiltration Level, Tumor Purity, and Stromal Content in PCa

The ESTIMATE method was used to assess the immune cell infiltration level, including immune score, tumor purity, and stromal content (stromal score) for each PCa sample in four datasets (23). The ABSOLUTE algorithm (24) was also used to evaluate the ploidy and purity score of each PCa sample in the TCGA dataset. The Kruskal–Wallis test was employed to test the difference between PCa subtypes.

### Comparison of the Proportions of Immune Cell Subsets Among PCa Subtypes

CIBERSORT algorithm (25) was used to infer the proportions of LM22 human immune cell subclasses. The 1,000 permutations and *P* < 0.05 was set as the criteria for inclusion of tumor samples. Total T cells were calculated as a sum of CD8+ T cells, CD4+ naïve T cells, CD4+ memory resting T cells, CD4+ memory activated T cells, follicular helper T cells, regulatory T cells (Tregs) and T cells gamma delta fractions between HIF-1_H and HIF-1_L subtypes. Total macrophage fraction was input as a sum of M0, M1, and M2 macrophage fractions. Total B cells were estimated as a sum of B cells memory and B cells naïve.

### Survival Analyses

We compared the disease-free survival (DFS) of PCa patients considering tumor subtypes. The Kaplan-Meier survival analysis was used to compare the differences among three PCa subclasses in Taylor and TCGA datasets, which have available survival data. The log-rank test was used to calculate the significance of survival time differences with a threshold of *P* < 0.05.

### Gene Set Enrichment Analysis (GSEA) and Gene Set Variation Analysis (GSVA)

Gene Set Enrichment Analysis (GSEA) and Gene Set Variation Analysis (GSVA) were conducted to determine the overall pathway of gene-set activity score for each sample in the Taylor and TCGA datasets (20). The Gene sets based on the c2/c5 curated signatures were downloaded from the Molecular Signature Database (MSigDB) of Broad Institute. KEGG pathways that were upregulated in HIF-1_H and HIF-1_L were then identified. The pathways that were significantly enriched were identified based on FDR < 0.05. The common pathways in both datasets were selected.

### Identification of PCa Subtype-Specific Networks

The WGCNA (26) method was used to identify gene modules that are significantly related to genes that are highly associated with immune cell infiltration, based on gene co-expression analysis using the TCGA dataset. The gene-gene interaction network was built using Cytoscape 3.3.2.

### Mutation Analysis

Mutation data in the MAF of PCa patients were used in the TCGA dataset for genetic and epigenetic analysis. The R package “maftools” was used to display the mutation profile of each subtype (27). The maftools was also used to impute the mutation rate of each gene and to identify significant mutant genes in the different subtypes (*P* < 0.05).

### Prediction for Chemo/Immunotherapeutic Response

Tumor immune dysfunction and exclusion (TIDE) algorithms (28) and subclass mapping (29) are used to predict clinical response to immune checkpoints between the HIF-1_H and HIF-1_L in the TCGA dataset. The chemotherapeutic response of each sample was predicted based on the largest publicly available pharmacogenomics database [Pharmaceutical Sensitivity Genomics in Cancer (GDSC), https://www.cancerrxgene.org/] (24). The prediction procedure was performed by the R software package “pRRophetic,” where the half-maximal inhibitory concentration (IC_50_) of the samples was demonstrated using ridge regression and the prediction accuracy was assessed using 10-fold cross-validation based on the GDSC training set (30).

### Identification of HIF-1a in HPA Dataset

HIF-1a is a core factor in the HIF signaling pathway. Here, immunohistochemistry (IHC) data from the Human Protein Atlas database (HPA, https://www.proteinatlas.org/) was used to determine the protein expression of HIF-1a between PCa and normal tissues (31).

### Statistical Analysis

All statistical tests were analyzed using R (3.5.2) utilizing a χ2 or Fisher's exact test for categorical data. A Wilcoxon test (Mann-Whitney test) and the Kruskal-Wallis test were used for two or more continuous data groups (32). Kaplan-Meier curve (33) was conducted to screen prognostic immune cell subclasses for survival data. Survival analysis was performed using the R package “survival.” Fisher's independence exact test is used to statistically classify the relationship between clinical information and defined subtypes. For all statistical analyses, *P* < 0.05 was considered statistically significant.

## Results

### Identification of Immunogenomic PCa Subtypes

**Figure 1:**
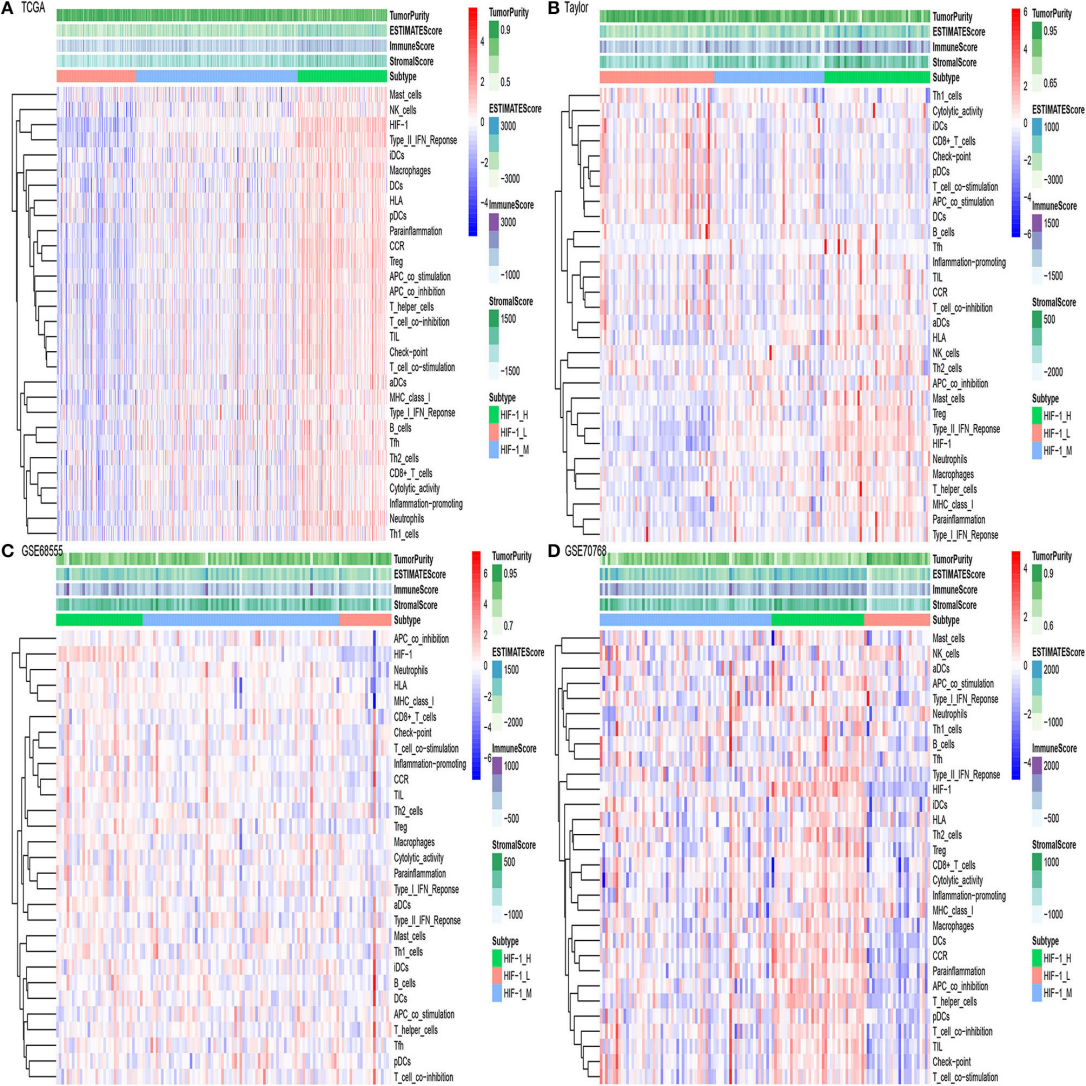
The hierarchical clustering of PCa exhibits three clusters in four different datasets. **(A–D)** TCGA PCa, Taylor, GSE68555, and GSE70768.

The flowchart was exhibited in Supplementary Figure 1. The gene set of the HIF-1 signaling pathway was downloaded from KEGG; a total of 109 genes were included in the KEGG pathway. Then, GSVA was performed to infer the enrichment scores across the PCa samples using the gene set. Unsupervised clustering analysis was conducted across the tumor samples in three PCa databases [Taylor (16), TCGA (17), and [GSE70768 (18) and GSE68555 (19)]]; all the four datasets showed similar clustering results, with three clusters separated. The three clusters: HIF-1 High (HIF-1_H), HIF-1 Medium (HIF-1_M), and HIF-1 Low (HIF-1_L) were defined (Figures 1A–D). The results demonstrate that immune cell infiltration increased with an increased enrichment score of the HIF-1 pathway in the TCGA dataset. A similar trend was observed in the GSE70768 and GSE68555 datasets, but the trend was not as distinct in the Taylor dataset.

**Figure 2:**
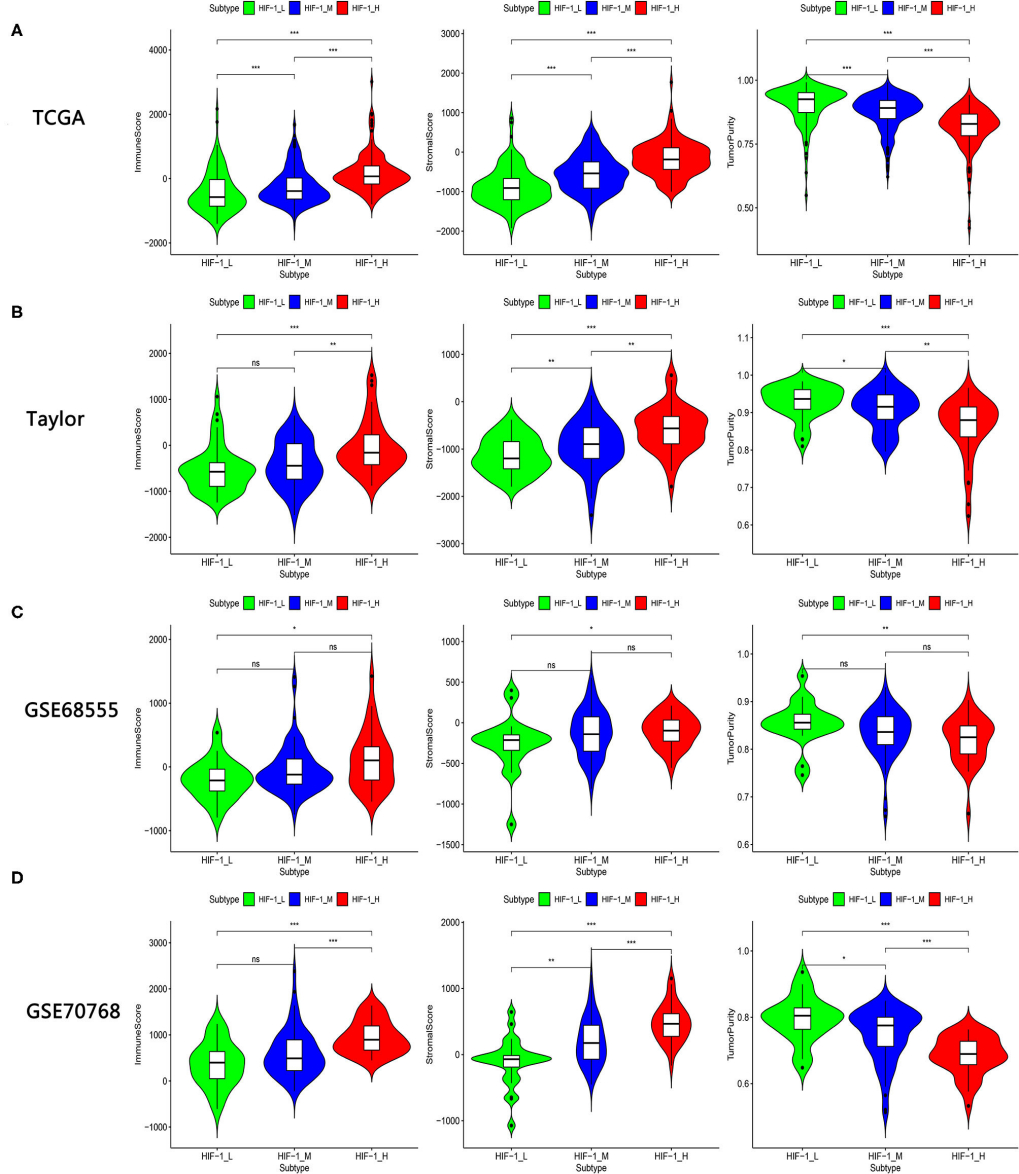
Comparison of the Stromal Score, Immune Score, and Tumor Purity among four PCa subtypes. **(A–D)** TCGA PCa, Taylor, GSE68555, and GSE70768. (*P < 0.05, **P < 0.01; ***P < 0.001; ns, P = 1).

The immune score was significantly higher in the HIF-1_H subset in all four data sets, whereas stromal score and tumor purity were significantly higher in the HIF-1_L subset (Kruskal–Wallis test, *P* < 0.05) (Figures 2A–D, Table 1). The ABSOLUTE algorithm showed that the purity score was lower in the HIF-1_H subset, for the TCGA dataset (Supplementary Figure 2). These results indicate that HIF-1_H contains the highest number of immune cells and stromal cells, while HIF-1_L contains the highest number of tumor cells.

**Table 1:** Comparison of the stromal score, immune score, and tumor purity among three PCa subtypes in four datasets.

**Dataset**	**Comparison**	**Immune score**	**Stromal score**	**Tumor purity**
		**(*P*-value)**	**(*P*-value)**	**(*P*-value)**
TCGA	HIF-1_H vs. HIF-1_L	6.40E−15	2.22E−16	2.22E−16
	HIF-1_H vs. HIF-1_M	7.40E−14	5.90E−14	4.20E−16
	HIF-1_M vs. HIF-1_L	0.00058	2.80E−10	2.90E−07
Taylor	HIF-1_H vs. HIF-1_L	3.40E−06	3.90E−08	1.90E−07
	HIF-1_H vs. HIF-1_M	0.004	0.0014	0.0013
	HIF-1_M vs. HIF-1_L	0.13	0.0095	0.03
GSE70768	HIF-1_H vs. HIF-1_L	3.70E−06	1.80E−09	8.70E−09
	HIF-1_H vs. HIF-1_M	4.70E−05	0.00011	2.30E−05
	HIF-1_M vs. HIF-1_L	0.1	0.0013	0.011
GSE68555	HIF-1_H vs. HIF-1_L	0.013	0.035	0.0076
	HIF-1_H vs. HIF-1_M	0.085	0.51	0.083
	HIF-1_M vs. HIF-1_L	0.13	0.098	0.077

**Figure 3:**
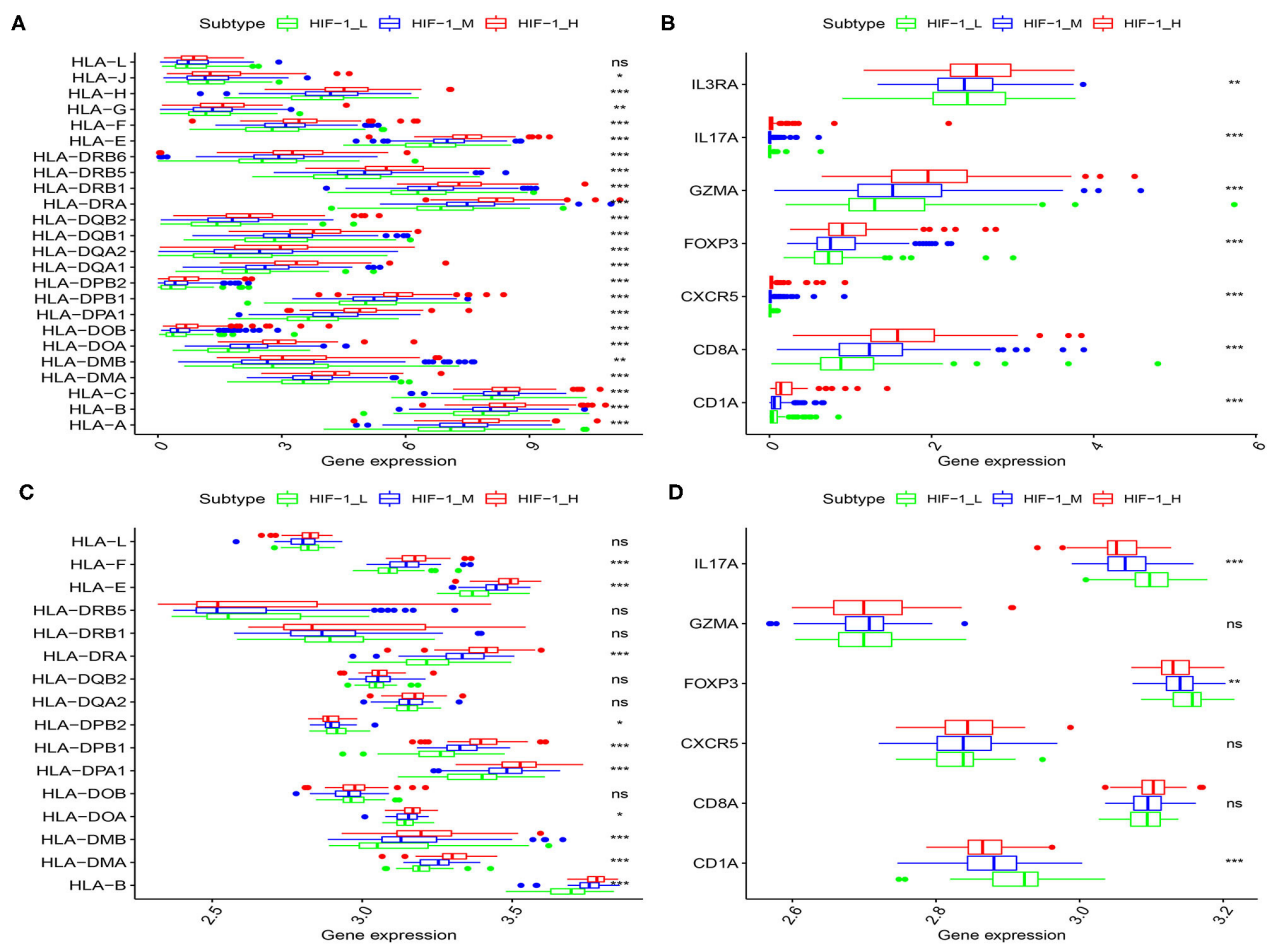
Comparison of the expression of HLA genes (**A,C**: TCGA and Taylor) and immune cell subpopulation marker genes (**B,D**: TCGA, and Taylor) among three PCa subtypes. (*P < 0.05, **P < 0.01; ***P < 0.001; ns, P = 1).

The human leukocyte antigen (HLA) complex is an important component of the immune system. It stimulates immune cells to provide protection and defense against cancer because tumor antigens must be presented in an HLA-restricted manner to be recognized by T cell receptors. In this study, HLA genes exhibited significantly higher expression in HIF-1_H and significantly lower expression in HIF-1_L (Kruskal-Wallis test, *P* < 0.05, Figures 3A,C). The expression levels of various immune cell marker genes such as CD8A (cytotoxic T cell), CXCR5 (Tfh cell), FOXP3 (Treg), IL-17 (Th17 cell), CD1A (iDC), and IL3RA (pDC) (34) were highest in HIF-1_H and the lowest in HIF-1_L (Figures 3B,D). This finding is consistent with the previous observation that the subtype HIF-1_H is enriched in immune cell type.

**Figure 4:**
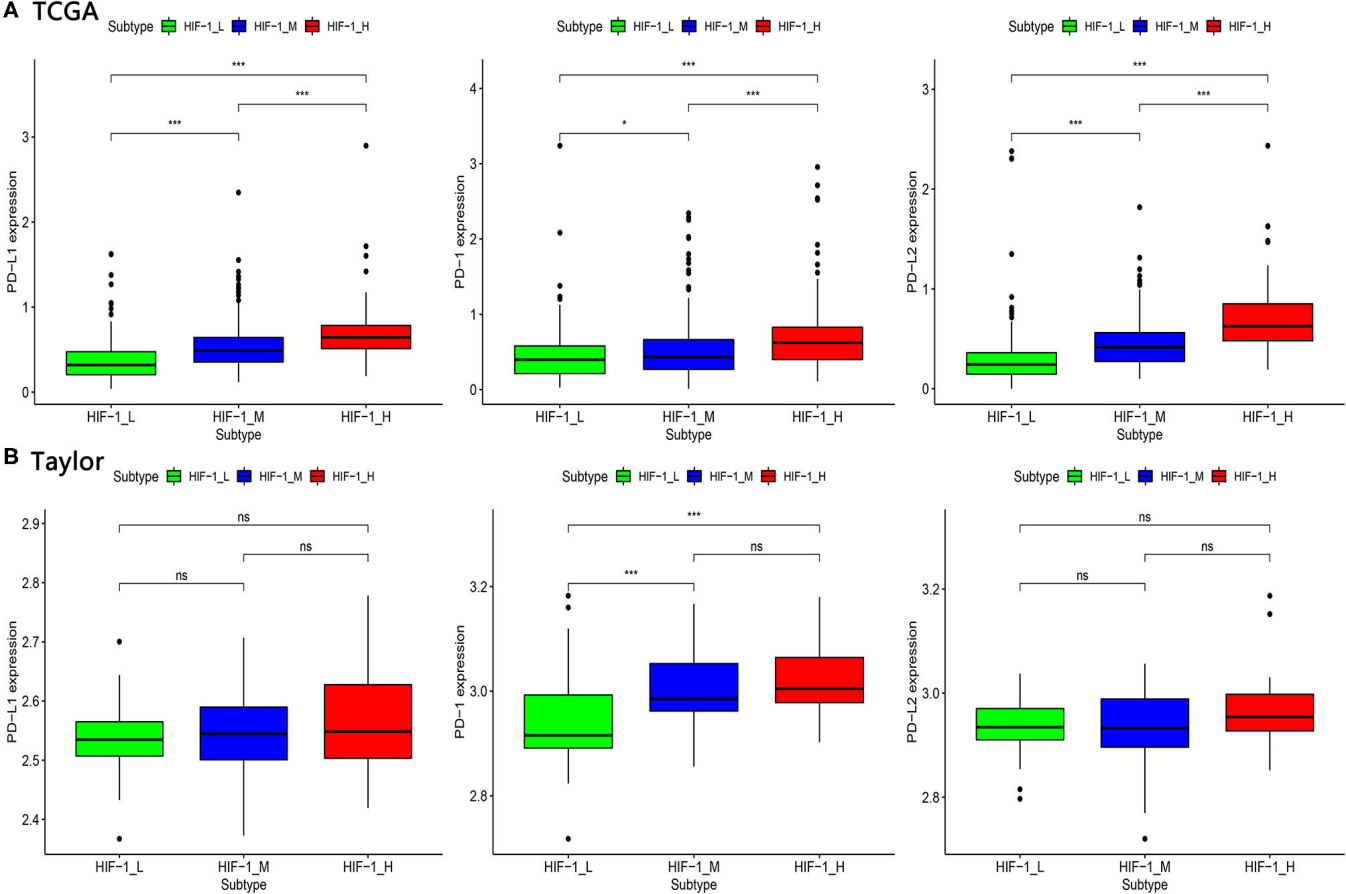
Comparison of the expression of PD-1, PD-L1, and PD-L2 among three PCa subtypes in TCGA **(A)** and Taylor **(B)** dataset. (*P < 0.05, **P < 0.01; ***P < 0.001; ns, P = 1).

The expression of PD-L1 (programmed death-ligand 1), PD1 (prephenate dehydratase 1), and PD-L2 (programmed death-ligand 2) for the three PCa subtypes were explored, in the four datasets. The results indicate that HIF-1_H exhibited the highest expression of PD-L1, PD1, and PD-L2, while HIF-1_L had the lowest expression of PD-L1, PD1, and PD-L2 (Kruskal–Wallis test, *P* < 0.05) (Figures 4A,B). This suggests that PCa subtype HIF-1_H may have a better response to anti-PD-L1 immunotherapy than other PCa subtypes because PD-1/PD-L1 expression is often positively correlated with immunotherapy response (35).

**Figure 5:**
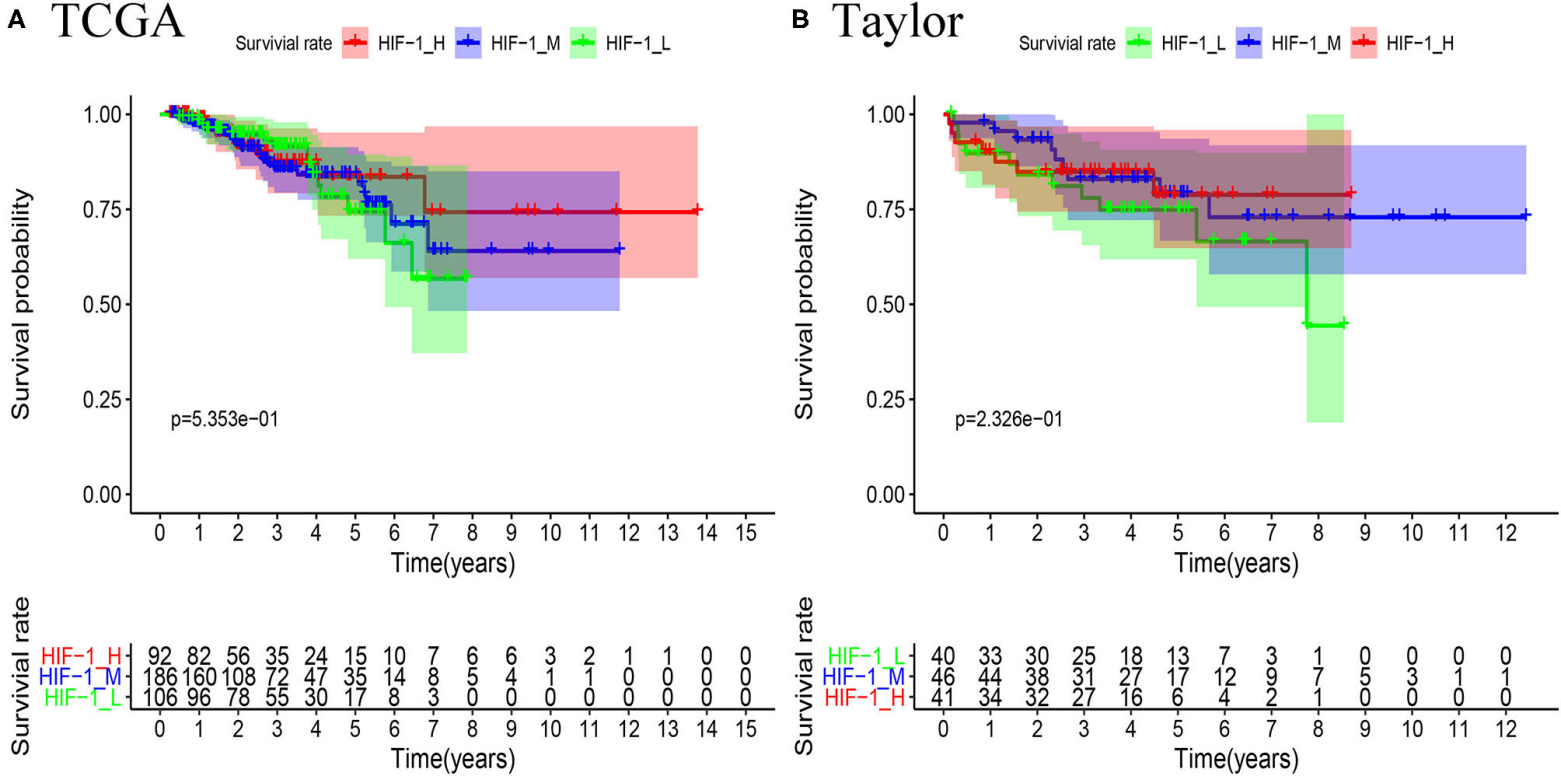
Comparison of RFS survival prognosis among three PCa subtypes in the TCGA PCa **(A)** and Taylor **(B)** datasets.

Survival analyses suggested that these PCa subsets have distinct clinical outcomes. The HIF-1_H subtype likely has a better survival prognosis than the HIF-1_M and HIF-1_L subtypes, but there was no significant survival difference between the HIF-1_M and the HIF-1_L subtypes (Figures 5A,B).

### Comparisons of the Proportions of Immune Cells and Clonal Heterogeneity Between PCa Subtypes

**Figure 6:**
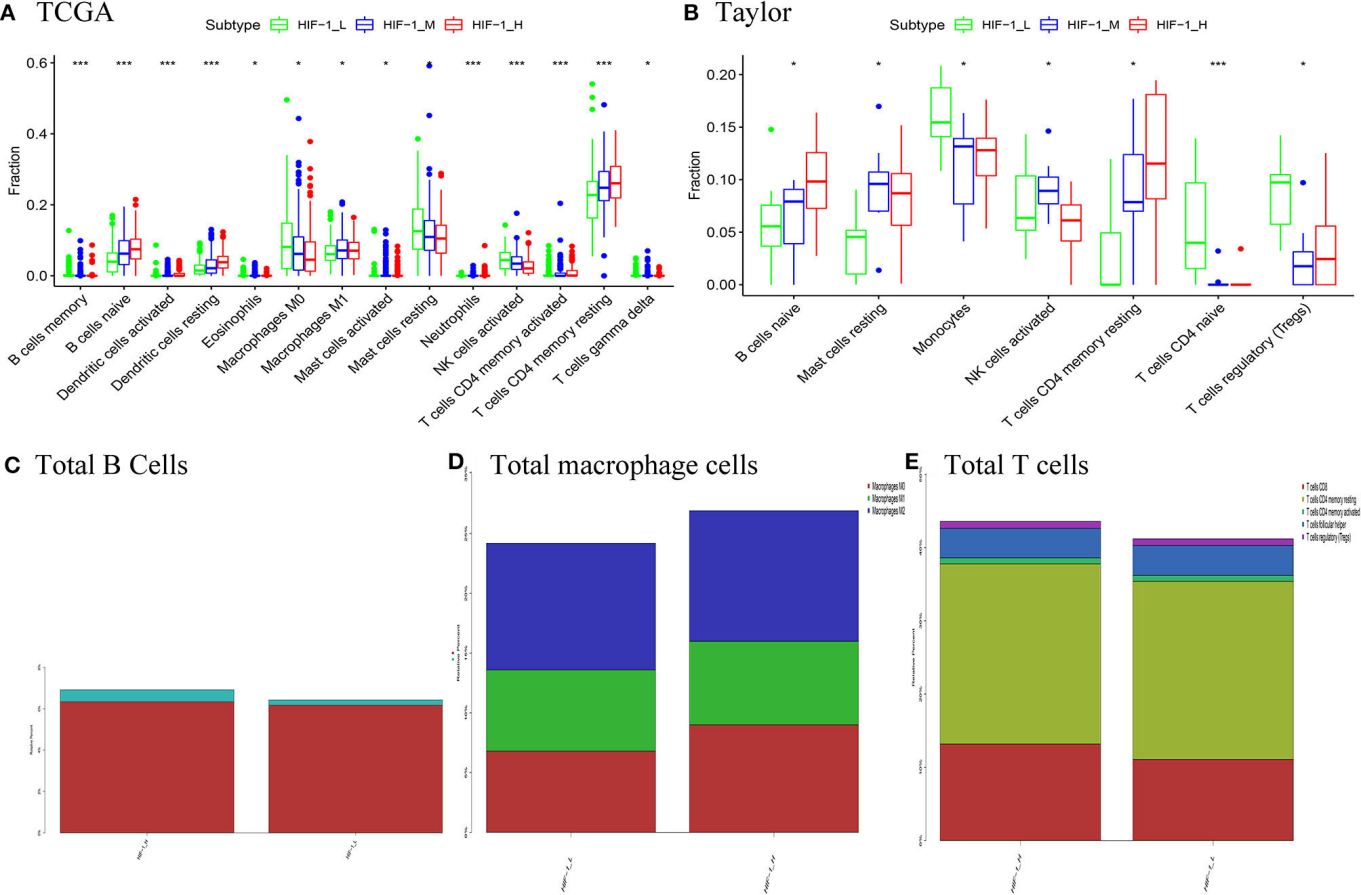
Comparison of the proportions of immune cell subsets among PCa subtypes in TCGA **(A)** and Taylor **(B)** dataset. Comparison of total B cells **(C)**, total Total macrophage cells **(D)**, and total T cells **(E)** among PCa subclasses in the TCGA PCa cohort. (*P < 0.05, **P < 0.01; ***P < 0.001; ns, P = 1).

CIBERSOFT algorithm was conducted to infer the landscape of tumor microenvironment (TME) cell infiltration between PCa subtypes in the TCGA dataset. The findings showed that 14/22 immune cells had significant difference among the PCa subtypes; B cells naïve, dendritic cells resting, T cells CD4 memory activated and T cells CD4 memory resting were significantly higher in the HIF-1_H subset, while Macrophages M1 and NK cells activated were significantly lower in the HIF-1_L subset in the TCGA dataset. In the Taylor dataset, Only 9/22 immune cells had significant difference among the PCa subtypes; B cells naïve and T cells CD4 memory resting were relatively higher in the HIF-1_H subset, monocytes and T cells regulatory (Tregs) were relatively higher in the HIF-1_L subset (Figures 6A,B). The fractions of total T cells, total B cells, and total Macrophages were higher in the HIF-1_H subset in the TCGA dataset (Figures 6C–E). This finding aligns with the previous observation that the subtype HIF-1_H is enriched with immune cells.

### Identification of PCa Subtype-Specific Pathways, Gene Ontology

**Figure 7:**
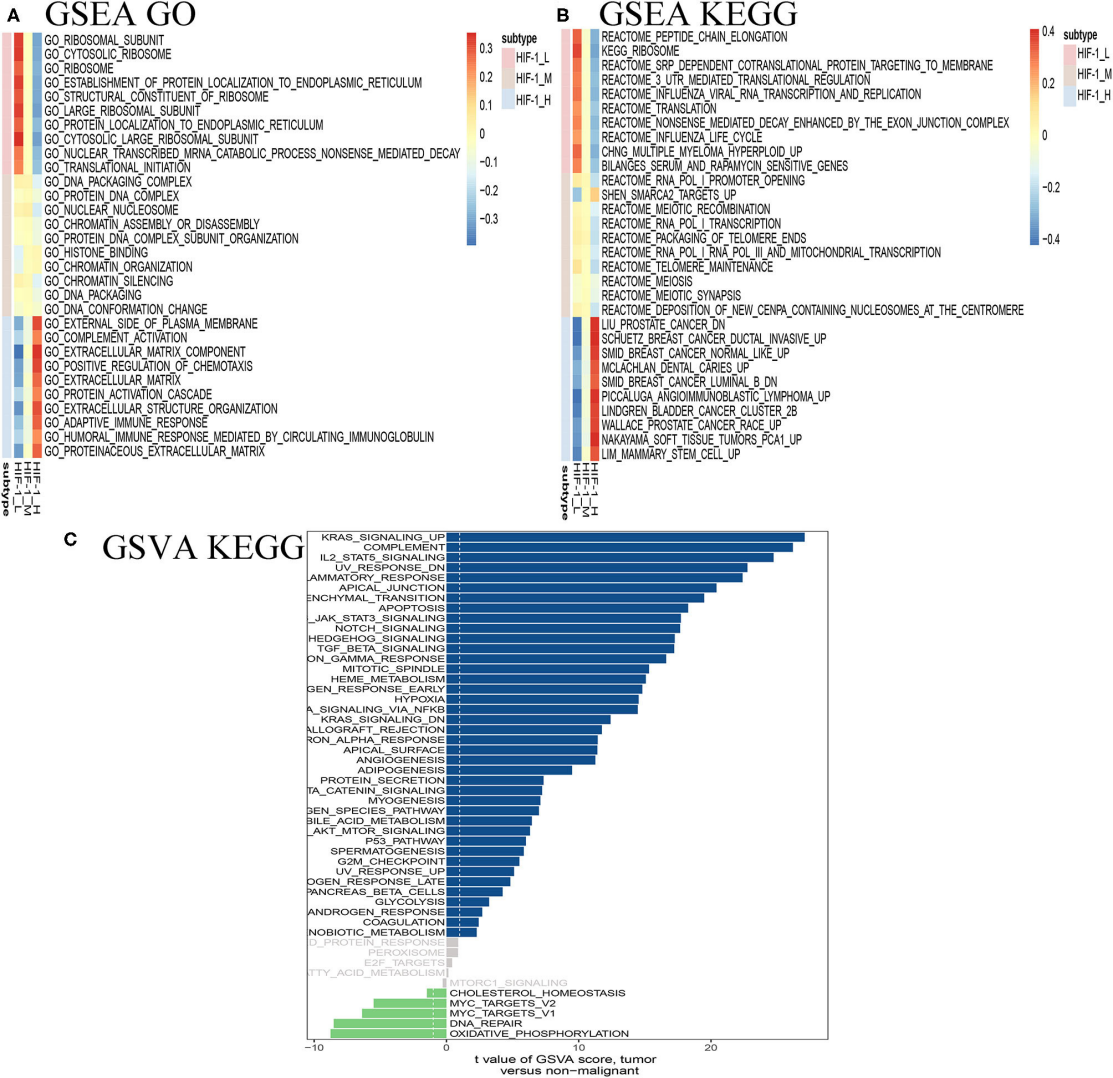
Identification of PCa subtype-specific up-regulated GO **(A)** and KEGG **(B)** among three PCa subtypes using the GSEA method in the TCGA dataset. **(C)** GSVA reveals the disparity in the KEGG pathway between the HIF-1_L and HIF-1_H subset in the TCGA dataset.

GSEA revealed distinct enriched up-regulated gene sets between the HIF-1_H and HIF-1_L (Figures 7A,B). Typically, the immune-related pathways were highly active in the HIF-1_H subclass. Several immune-related GO terms were identified in the HIF-1_H subtypes, including B cell receptor signaling pathway, T cell differentiation, and B cell-mediated immunity. The HIF-1_H subtypes were enriched in the cell cycle, cell repair, cell adhesion, adherens junction function, including ribosome, RNA binding, and cellular protein complex disassembly. Compared with the HIF-1_L subtype, adaptive immune response, and humoral immune response mediated by circulating immunoglobulin, the extracellular matrix were highly activated. In contrast, cytosolic ribosome and translational initiation were activated in the HIF-1_L subtype. In terms of the KEGG pathway, the immune-related pathways were highly activated in the HIF-1_H subtype and included Th1 and Th2 cell differentiation, leukocyte trans-endothelial migration, and B cell and T cell receptor signaling pathways. The findings validated that elevated immune activity is in the HIF-1_H subtype. Besides, multiple cancer-related pathways identified were hyperactivated in HIF-1_H, TNF signaling pathway, PI3K-Akt signaling pathway, prostate cancer, and Wallace prostate cancer race. In contrast, HIF-1_L was mainly enriched in pathways related to peptide chain elongation, ribosome, and influenza life cycle.

The GSVA analysis revealed similar results. KRAS signaling, IL2 stat5 signaling, and epithelial-mesenchymal transition were highly activated in the HIF-1_H, while DNA repair and oxidative phosphorylation were hyperactivated in HIF-1_L (Figure 7C).

### Clinical Feature of PCa Subtypes

**Figure 8:**
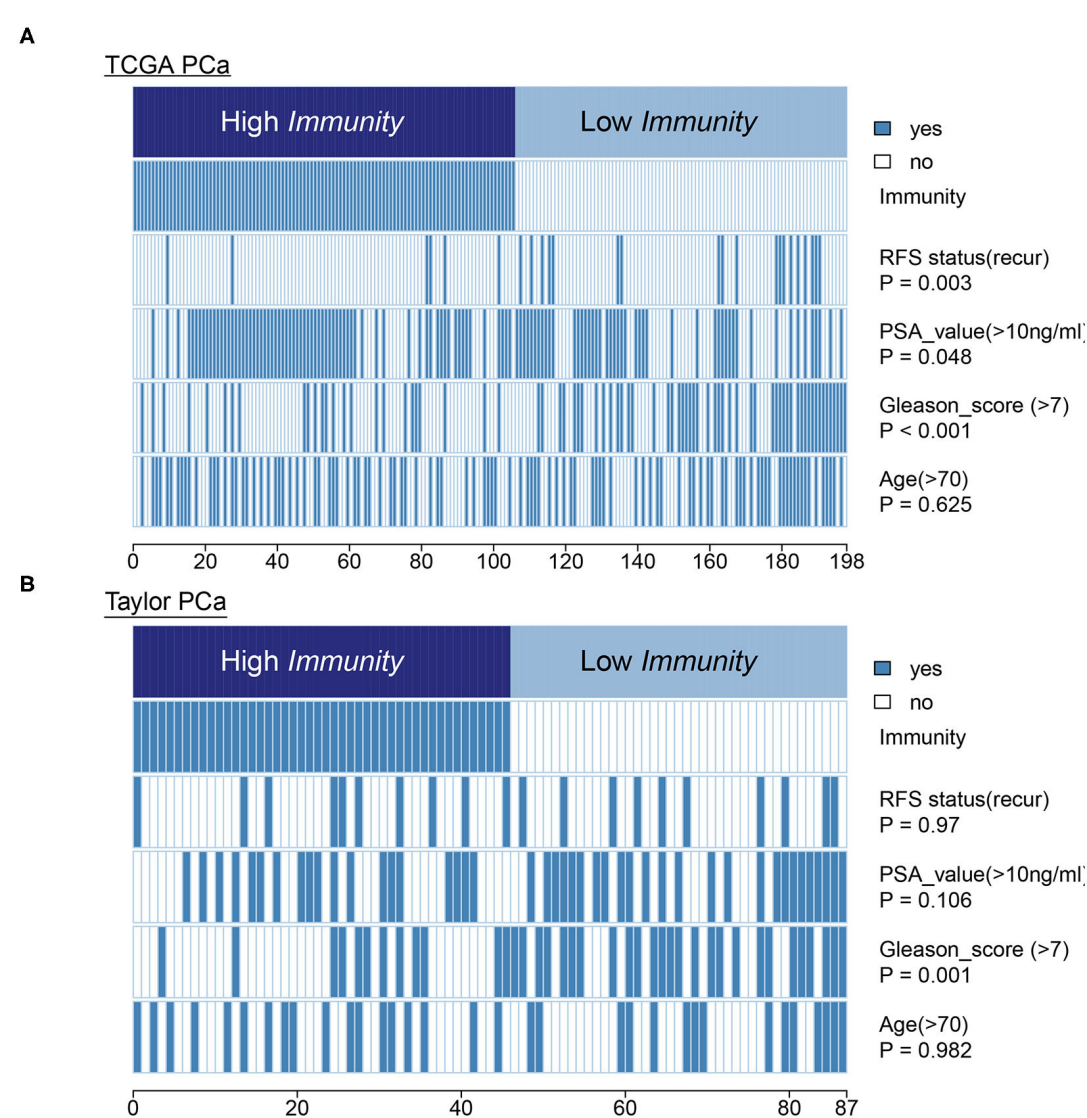
Comparing PSA value, Gleason score, and age between the immune-H and immune-L PCa subsets. Statistical significance was performed using the Chi-square test. The heatmap illustrates the association of different clinical characters with PCa subsets. **(A,B)** TCGA PCa and Taylor, respectively.

In terms of clinical features, HIF-1_H had a lower Gleason score and PSA level compared to HIF-1_L in the TCGA PCa cohort. The heatmap illustrates the association of the different clinical characters between the two subgroups. However, there was no difference in RFS status and age between the two subtypes (Figure 8A). In the Taylor dataset, HIF-1_H had a lower Gleason score (Figure 8B). There was no difference in the RFS status, age, and PSA values between the two subtypes. Statistical significance was determined using the Fish's exact test.

### Identification of PCa Subtype-Specific Network and Hub Genes

**Figure 9:**
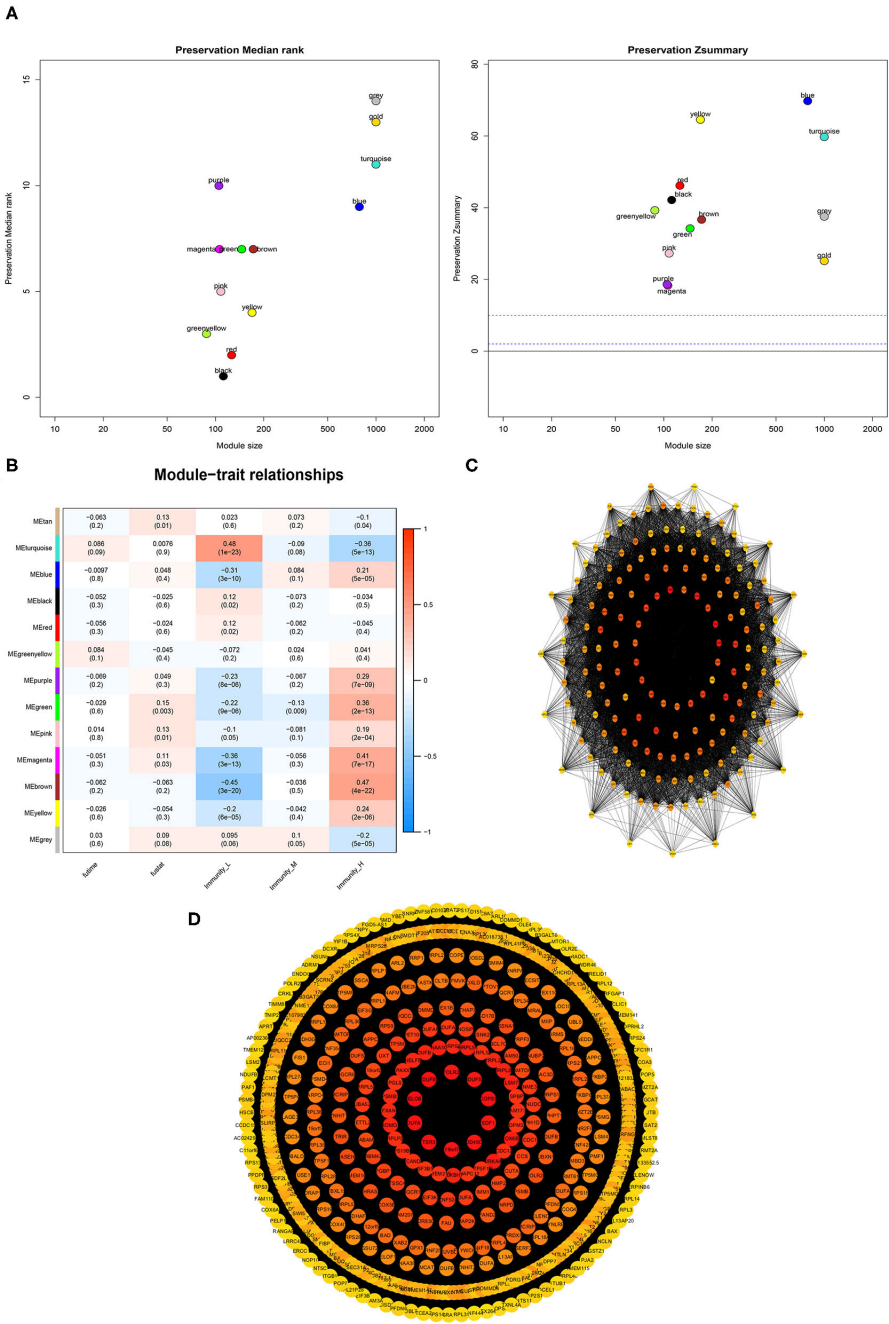
Association between clinical features and PCa subtypes using WGCNA analysis. **(A)** The median Rank and Zsummary statistics of the module preservation. **(B)** The module-feature associations among three PCa subsets. **(C,D)** The protein-protein interaction (PPI) network as constructed in the Brown and Turquoise modules, respectively.

A weighted gene co-expression network analysis of the TCGA dataset was conducted using the WGCNA method. Module preservation analysis demonstrated that 13 modules were the most stable with Zsummary statistics >10. Several gene modules that were significantly different based on PCa subtype, survival time, or survival status were identified (Figure 9A). The turquoise and magenta modules were negatively associated with the HIF-1_H subtype, while brown and yellow modules were positively correlated with HIF-1_H, especially the brown module. The opposite trend was observed in the HIF-1_L subtype (Figure 9B). A weighted co-expression network from the brown and turquoise modules was constructed (Figures 9C,D).

### Comparisons of Gene Mutation Between PCa Subtypes

**Figure 10:**
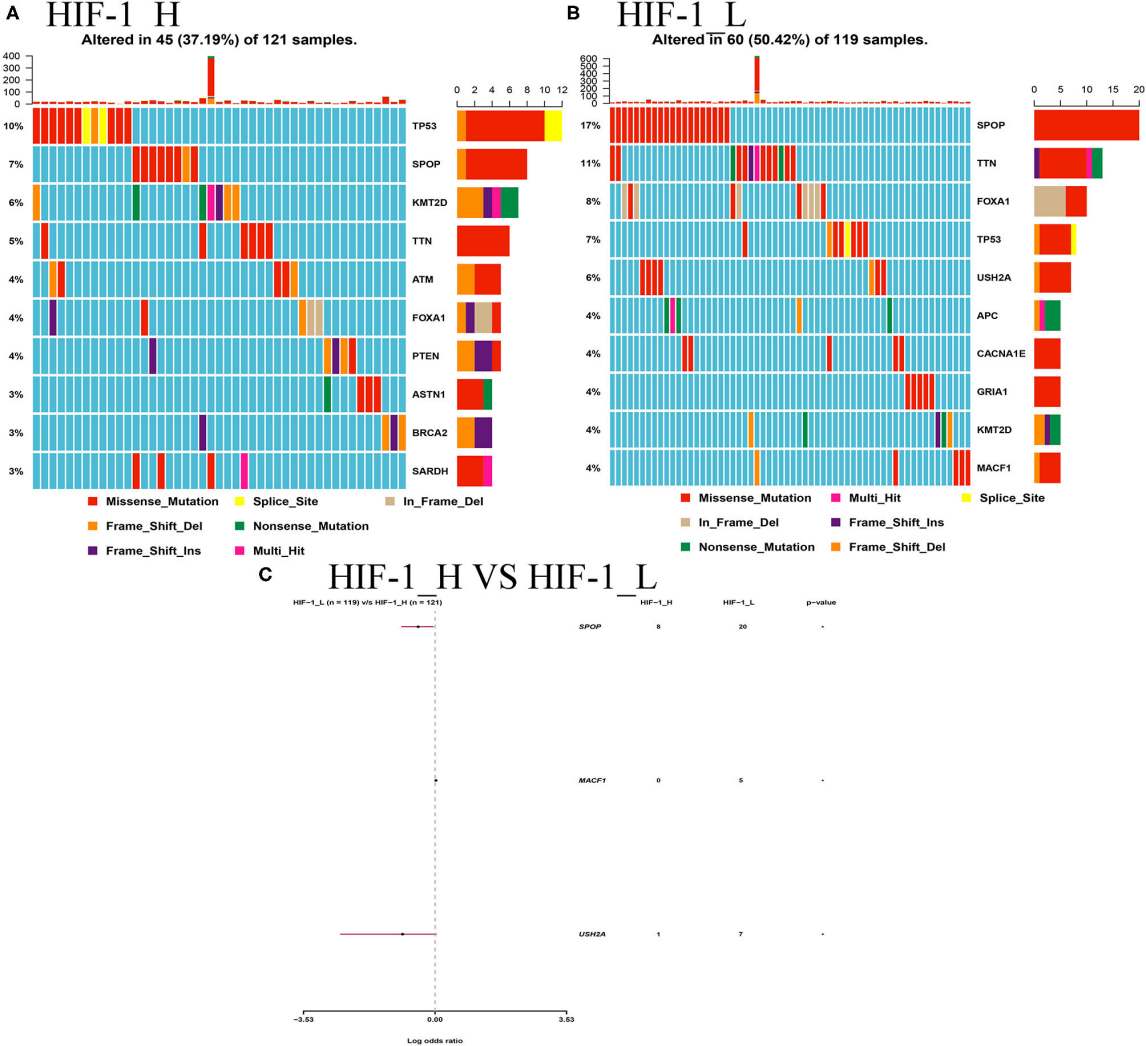
**(A)** Mutation analysis between the HIF-1_L and HIF-1_H subsets in the TCGA dataset. **(B)** Gene mutation profiles of highly mutated genes among the two subtypes. **(C)** The forest plots show the comparison results of gene mutations (*P < 0.05, **P < 0.01; ns, P = 1).

This study examined the association between the HIF-1_H/L subtypes and somatic mutation count. Highly mutated gene profiles are shown in Figures 10A,B. The most mutations in the HIF-1_H subtype were found in the TP53, PTEN, and BRCA2 genes, whereas the HIF-1_L subtype had the most mutations in the SPOP, FOXA1, and TP53 genes. SPOP and USH2A genes exhibited a higher mutation rate in the HIF-1_H subtype, and MACF1 exhibited a higher mutation rate in the HIF-1_L subtype with the cut-off point < 0.05 (Figure 10C).

### Prediction for Response to Immunotherapy or Anti-cancer Drug in PCa Subtypes

**Figure 11:**
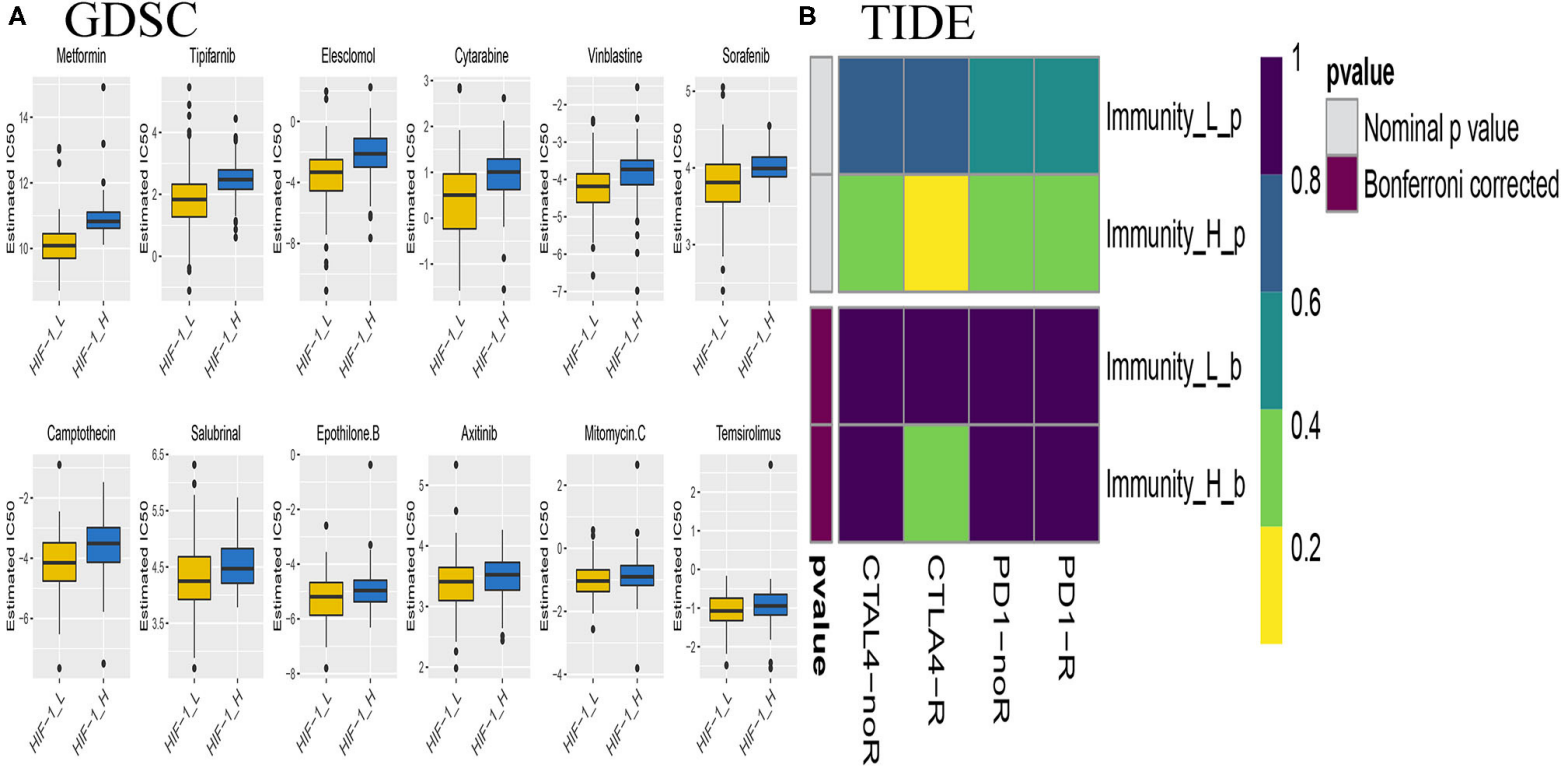
Differential putative chemotherapeutic and immunotherapeutic response. The box plots of the estimated IC_50_ for chemotherapeutic drugs are shown: **(A)** for Immune-H and Immune-L PCa subsets, **(B)** Submap analysis manifested that Immune-H could be more sensitive to the immunotherapy (Bonferroni-corrected *P* < 0.05).

The submap algorithm was used to predict the likelihood of responding to immunotherapy in the TCGA PCa cohort, although immunological checkpoint drugs have not been approved for conventional use in PCa. The analysis showed that HIF-1_H was likely to respond better to immunotherapy than HIF-1_L (*P* = 0.04). For the TIDE prediction, a subclass mapping method was used to compare the expression profiles of the three PCa subtypes with another published data set containing 47 melanoma patients who responded to immunotherapy. The results showed that HIF-1_H was the most promising subtype for CTLA4 treatment (Bonferroni correction *P* < 0.05) (Figure 11A).

Chemotherapy is a common treatment for PCa. The response of the three subtypes to commonly used drugs was evaluated. The prediction model on the GDSC cell line dataset was trained by ridge regression. Satisfactory prediction accuracy was evaluated by 10-fold cross-validation for the TCGA PCa cohort. The IC_50_ for each sample in the TCGA dataset was estimated based on the predictive model of chemo drugs; there were significant differences in the estimated IC_50_ against HIF-1_H, for several drugs. HIF-1_H may be more sensitive to commonly used chemotherapy (ABT 888, Temsirolimus, and EHT 1864, *P* < 0.05) (Figure 11B).

### Immunohistochemistry Verification of HIF-1a in HPA Database

**Figure 12:**
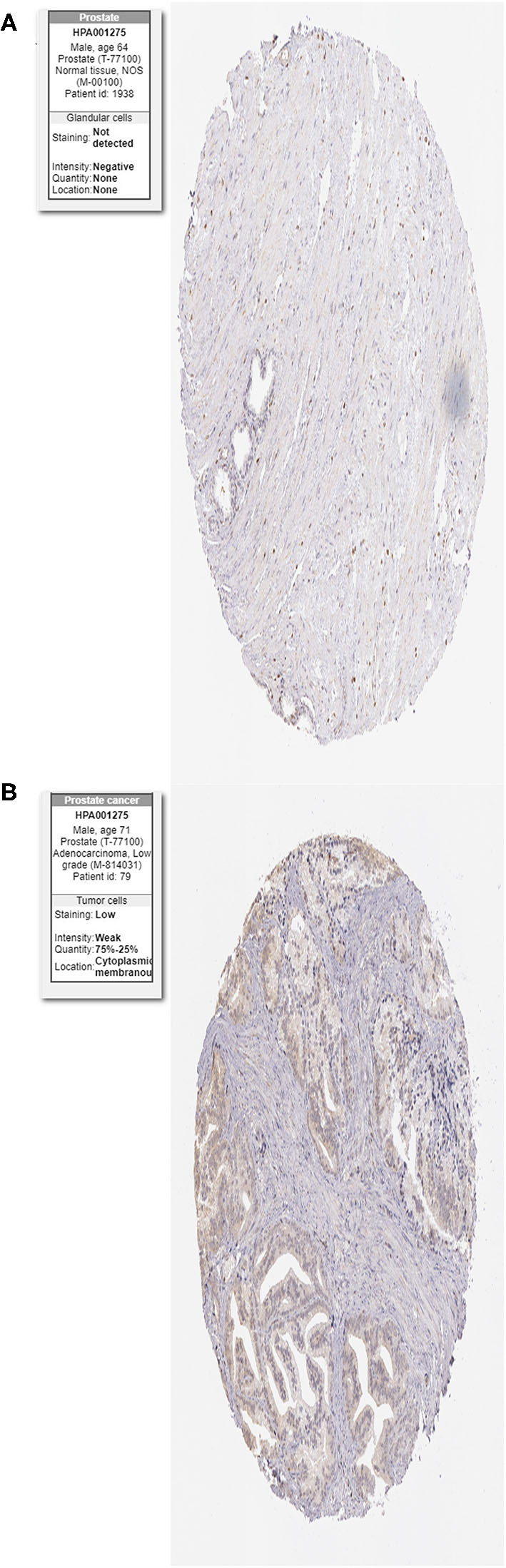
Immunohistochemistry of the HIF-1a in PCa and normal tissues from the human protein atlas (HPA) database. **(A)** Protein levels of HIF-1a in normal prostate tissue, **(B)** Protein levels of HIF-1a in PCa tissues.

The protein levels of the HIF-1a were significantly higher in tumor tissues compared with normal tissues based on the HPA database (Figures 12A,B).

## Discussion

Prostate cancer remains one of the major tumors threatening male human health all worldwide (36). Prostate cancer therapy includes surgery, radiotherapy, chemotherapy, immunotherapy, and targeted approaches using antiangiogenic monoclonal antibodies and tyrosine kinase inhibitors, if tumors harbor a specific mutation. These modalities have provided therapeutic options, but the prognosis of advanced PCa is still not optimistic; the 5-year overall survival remains low. Prostate cancer causes a hypoxic environment due to the rapidly proliferating cells, structural and functional abnormalities of the tumor vasculature. Increased synthesis and decreased degradation of HIF-1a protein have been observed in PCa (37–39); HIF-1a is expected to be a feasible target considering the disease's insensitivity to immunotherapy.

In this study, the immunogenomic PCa subsets were classified based on enrichment scores and the HIF-1 signaling pathway using the publicly available four PCa datasets. Our results showed that PCa could be classified into three subtypes: HIF-1-H, HIF-1_M, and HIF-1_L. These results were validated in three other datasets; this classification was reproducible and predictable. Details of the subpopulation of the three subtypes in PCa were also revealed. HIF-1_H was enriched where there were greater immune cell infiltration and higher HIF enrichment score, and exhibited a better survival prognosis, whereas HIF-1-_L had higher tumor purity and stromal score. In the functional enrichment analysis, HIF-1_H had many subtype-specific pathways, including apoptosis, TNF signaling pathway, PI3K-Akt signaling pathway, prostate cancer Th1 and Th2 cell differentiation, Leukocyte trans-endothelial migration, B cell receptor signaling pathway, and T cell receptor signaling pathway. In contrast, the HIF-1_L subtype was enriched in the ribosome, cell cycle and cell repair, cell adhesion, and adherens junction function. The HIF pathway may trigger a hyperactivated immune-related pathway, which may be involved in the pathogenesis of cancer. For example, HIF-1α exerts important functional roles in both innate and adaptive immune cells, including macrophages (40), neutrophils (41), dendritic cells (42), and lymphocytes (43). It is also is an essential regulator of effector T cells responses in the tumor microenvironment (44).

HIF-1_H has a better prognosis, higher HIF-1 pathway enrichment scores, and lower Gleason score and PSA level than HIF-1_L. The submap and TIDE analysis suggested that HIF-1_H was more promising for CTLA4 treatment. Using the GDSC database, we deduced that HIF-1_H could be more sensitive to commonly used chemotherapies than HIF-1_L. The above implies that Cluster I may benefit from the combination of chemotherapy and immunotherapy (ABT 888, Temsirolimus, and EHT 1864, *P* < 0.05). Barreto-Andrade et al. (45) found that veliparib (ABT-888) can enhance the response of prostate cancer cells and tumors to ionizing radiation (IR). In a single-arm, open-label, pilot study, oral PARP inhibitor veliparib and the combination [veliparib and temozolomide (TMZ)] were observed to have antitumor activity in patients with metastatic castration-resistant prostate cancer (mCRPC) (46). Several studies demonstrate that Temsirolimus maintenance therapy is a potential treatment option for castration-resistant PCa (47–49). Comstock et al. (50) using human PCa models and primary tumors, showed that PD-0332991 (a potent and selective CDK4/6 inhibitor) exerts antitumor properties. These findings indicate that HIF-1a is a major regulator of cellular responses to the hypoxic microenvironment, is elevated in prostate cancer, and is considered a viable target in the treatment of prostate cancer.

## Conclusions

The identification of immunogenomic PCa subtypes based on the HIF-1 signaling pathway has potential clinical implications for PCa treatment. Immunopositive PCa subtypes may help to solve the poor response of PCa to immunotherapy; it is expected that immunotherapy will be used in the personalized treatment of PCa patients.

## Data Availability Statement

The datasets presented in this study can be found in online repositories. The names of the repository/repositories and accession number(s) can be found in the article/Supplementary Material.

## Ethics Statement

The studies involving human participants were reviewed and approved by Guizhou Provincial People's Hospital. The patients/participants provided their written informed consent to participate in this study. Written informed consent was obtained from the individual(s) for the publication of any potentially identifiable images or data included in this article.

## Author Contributions

JS, JZ, WC, and GZ wrote the main manuscript text. JS prepared Figures 1–12. JS and JZ contributed to data analysis. All authors reviewed the manuscript.

## Conflict of Interest

The authors declare that the research was conducted in the absence of any commercial or financial relationships that could be construed as a potential conflict of interest.

## Supplementary Material

Supplementary Figure 1 The flowchart of data procession.

Supplementary Figure 2 Comparison of the expression levels of Immune cell subpopulation marker genes between three PCa subtypes.

Supplementary Table 1 The gene set of HIF-1 signaling pathway from KEGG dataset.
