# Supplementary figures and images for: Immunogenomic Profiling and Classification of Prostate Cancer Based on HIF-1 Signaling Pathway
Source: Front Oncol. 2020 Aug 6;10:1374. doi: 10.3389/fonc.2020.01374 (PMC7425731; doi:10.3389/fonc.2020.01374)

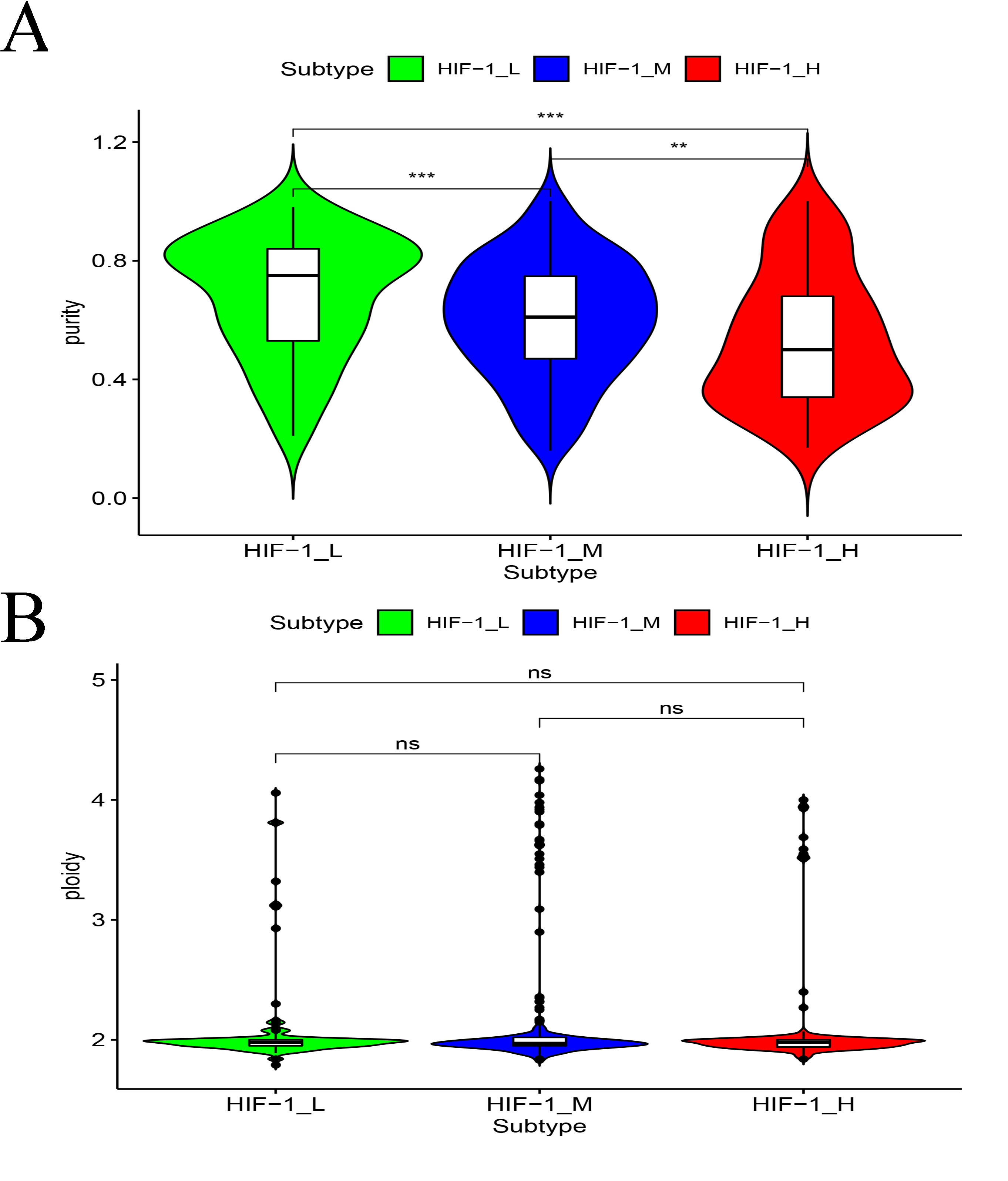

Supplement: Supplementary Figure 1 — The flowchart of data procession. [file Image_1.TIF]

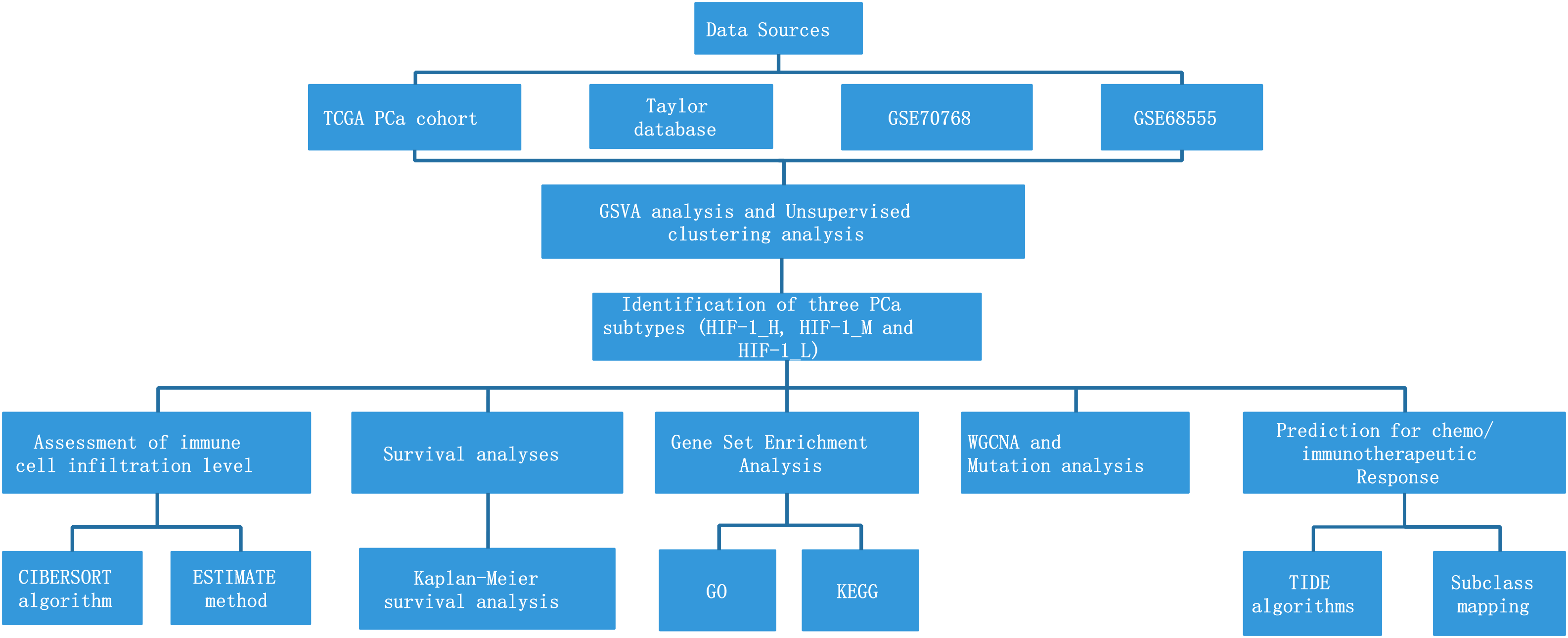

Supplement: Supplementary Figure 2 — Comparison of the expression levels of Immune cell subpopulation marker genes between three PCa subtypes. [file Image_2.TIF]
