# Supplementary material for: Immunogenomic Profiling and Classification of Prostate Cancer Based on HIF-1 Signaling Pathway
Source: Front Oncol. 2020 Aug 6;10:1374. doi: 10.3389/fonc.2020.01374 (PMC7425731; doi:10.3389/fonc.2020.01374)
Supplement: Supplementary Table 1 — The gene set of HIF-1 signaling pathway from KEGG dataset. [file Table_1.docx]

Supplementary Table 1: The gene set of HIF-1 signaling pathway from KEGG dataset.

| **gene symbol** |
| --- |
| IL6 |
| IL6R |
| STAT3 |
| TLR4 |
| IFNG |
| IFNGR1 |
| IFNGR2 |
| RELA |
| NFKB1 |
| INS |
| EGF |
| IGF1 |
| INSR |
| EGFR |
| IGF1R |
| ERBB2 |
| MAP2K1 |
| MAP2K2 |
| MAPK1 |
| MAPK3 |
| MKNK1 |
| MKNK2 |
| PIK3CA |
| PIK3CD |
| PIK3CB |
| PIK3R1 |
| PIK3R2 |
| PIK3R3 |
| AKT1 |
| AKT2 |
| AKT3 |
| MTOR |
| EIF4EBP1 |
| EIF4E |
| EIF4E2 |
| EIF4E1B |
| RPS6KB1 |
| RPS6KB2 |
| RPS6 |
| HIF1A |
| VHL |
| RBX1 |
| ELOC |
